# Field-Specific Intensity-modulated Proton Therapy Optimization Technique for Breast Cancer Patients with Tissue Expanders Containing Metal Ports

**DOI:** 10.7759/cureus.1698

**Published:** 2017-09-18

**Authors:** Maura Kirk, Gary Freedman, Thorsten Ostrander, Lei Dong

**Affiliations:** 1 Department of Radiation Oncology, Perelman School of Medicine, University of Pennsylvania; 2 Radiation Oncology, Scripps Proton Therapy Center

**Keywords:** pencil beam scanning proton therapy, intensity modulated proton therapy (impt), breast radiotherapy, proton pencil beam scanning left breast planning

## Abstract

This report aims to propose and present an evaluation of a robust pencil beam scanning proton multi-field optimized treatment planning technique for postmastectomy radiation of breast cancer patients with implanted tissue expanders containing an internal metal port. Field-specific split targets were created for optimization to prevent spots from traveling through the metal port, while providing uniform coverage of the target with the use of a multi-field intensity modulated optimization approach. Two beam angles were strategically selected to provide complementary target coverage and plan robustness. The plan was compared with an independently developed photon plan and evaluated for robustness with respect to isocenter shifts, range shifts, and variation of the water-equivalent thickness of the port. The proton plan resulted in clinically acceptable target coverage and dosage to neighboring normal tissues. The D95% coverage was 95.3% in the nominal proton plan, with a worst-case coverage of 90.1% (when considering 0.3 cm isocenter shifts combined with 3.5% range uncertainty), and the coverage varied less than 1% under a hypothetically extreme variation of the port density. The proton plan had improved dose homogeneity compared with the photon plan, and reduced ipsilateral lung and mean heart doses. We demonstrated that a practical, field-specific intensity-modulated proton therapy (IMPT) optimization technique can be used to deal with the challenge of metal port in breast cancer patients with tissue expanders. The resulting proton plan has superior dosimetric characteristics over the best-case scenario photon plan, and is also robust to setup and proton range uncertainties.

## Introduction

Clinical use of proton therapy has been rapidly expanding in recent years, according to surveys of the PTCOG, or Particle Therapy Co-Operative Group (https://www.ptcog.ch/index.php/ptcog-patient-statistics). The application of proton therapy to a larger range of treatment sites that are conventionally treated with photon or electron therapy (such as breast cancer), is being investigated through dosimetric studies and clinical trials, such as the RADCOMP randomized trial of photon versus proton therapy for patients with non-metastatic breast cancer. Some studies have suggested that treatment with protons may reduce the risk of cardiac toxicity to breast cancer survivors [1-3]. Proton therapy may be especially beneficial for patients indicated for postmastectomy radiation therapy (PMRT) after reconstruction, due to the technical challenges of their anatomy [4].

**Figure 1:**
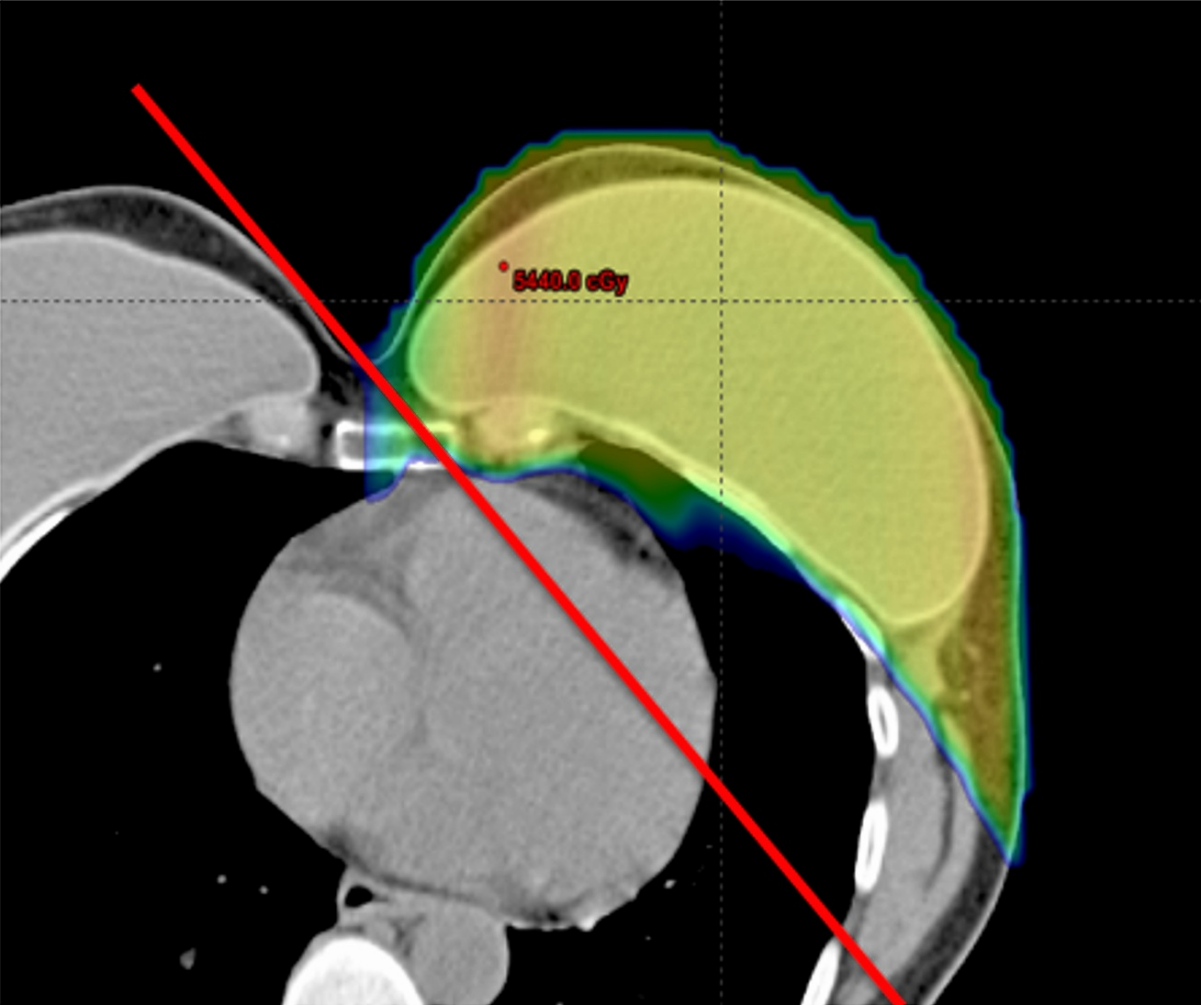
An example proton dose distribution for a case where the challenging anatomical geometry would produce unacceptable heart and lung doses if photon tangents (indicated by the red line) were used for planning.

However, conventional proton therapy with passive scatter technology reported high skin toxicities, while newer proton therapy with pencil beam scanning technology demonstrated superior dosimetric benefits [5]. The fundamental challenge with proton therapy is the range uncertainty [6]. This is particularly challenging when metal implants are involved. Breast implants include a highly convex shape, often very close to midline or the opposite breast, which causes the required fields for conventional tangential photon radiation to be associated with high lung or heart dose (Figure 1).

A common practice has been to deflate implants that cause anatomical difficulties prior to photon radiation, but this is not optimal for the patient. The optimal timing of radiation and reconstruction with tissue expanders containing metal ports versus permanent implants is unresolved, but recent studies have shown photon radiation to expanders associated with an increased risk for major complications [7-8]. However, in many cases, it remains clinically necessary to radiate the tissue expander that has a metallic port used for implant filling.

Proton therapy has potential to improve radiation dose delivery to these tissue expanders, remove the need for their deflation or removal prior to radiation, counter the often-associated increased heart and lung dose they cause with photon tangents, and reduce complications. The port presents an issue for treatment with protons because the range of the proton beam is sensitive to uncertainties caused by high density objects. Uncertainty in beam range could translate to a degradation of target coverage or loss of normal tissue sparing. One proposed method of handling the metallic port while treating with intensity modulated proton therapy (IMPT) shows promising dosimetric results, but requires in-depth measurement and Monte Carlo verification based on the specific construction of the port [9]. The purpose of this study was to design and investigate the robustness of a proton treatment technique that could be applied to any breast cancer patient with an implanted breast expander containing a metal port.

## Technical report

Patient selection

Although the goal of this study was to investigate a novel planning technique, the test case was from a well-known institution on the West Coast with only photon therapy equipment, a typical referral pattern for patients who come to the proton therapy center. The patient’s family and referring physician requested to evaluate the potential benefit of proton therapy for a left-side breast cancer patient post mastectomy. The patient's computed tomography (CT) scan was done at the photon therapy center, and simulated to receive photon therapy with deep inspiration breath-hold treatment. The CT scan had 2 mm slice thickness with the scan length extending from the chin to abdomen. The patient was immobilized in a Vac-Lok™ bag, with their ipsilateral arm raised above their head, and their head facing away from the side to be treated. Left-sided chestwall, axillary node, supraclavicular node, and internal mammary node (IMN) clinical target volumes (CTVs) were delineated according to the Radiation Therapy Oncology Group atlas, with the chest wall volume limited from extension into the ribs and intercostal muscles. Institutional constraints for target coverage and OAR doses are summarized in Table 1, with the plan prescribed to 50.4 Gy in 28 fractions to the chestwall and nodal volumes.

Handling metal port and image artifacts

**Figure 2:**
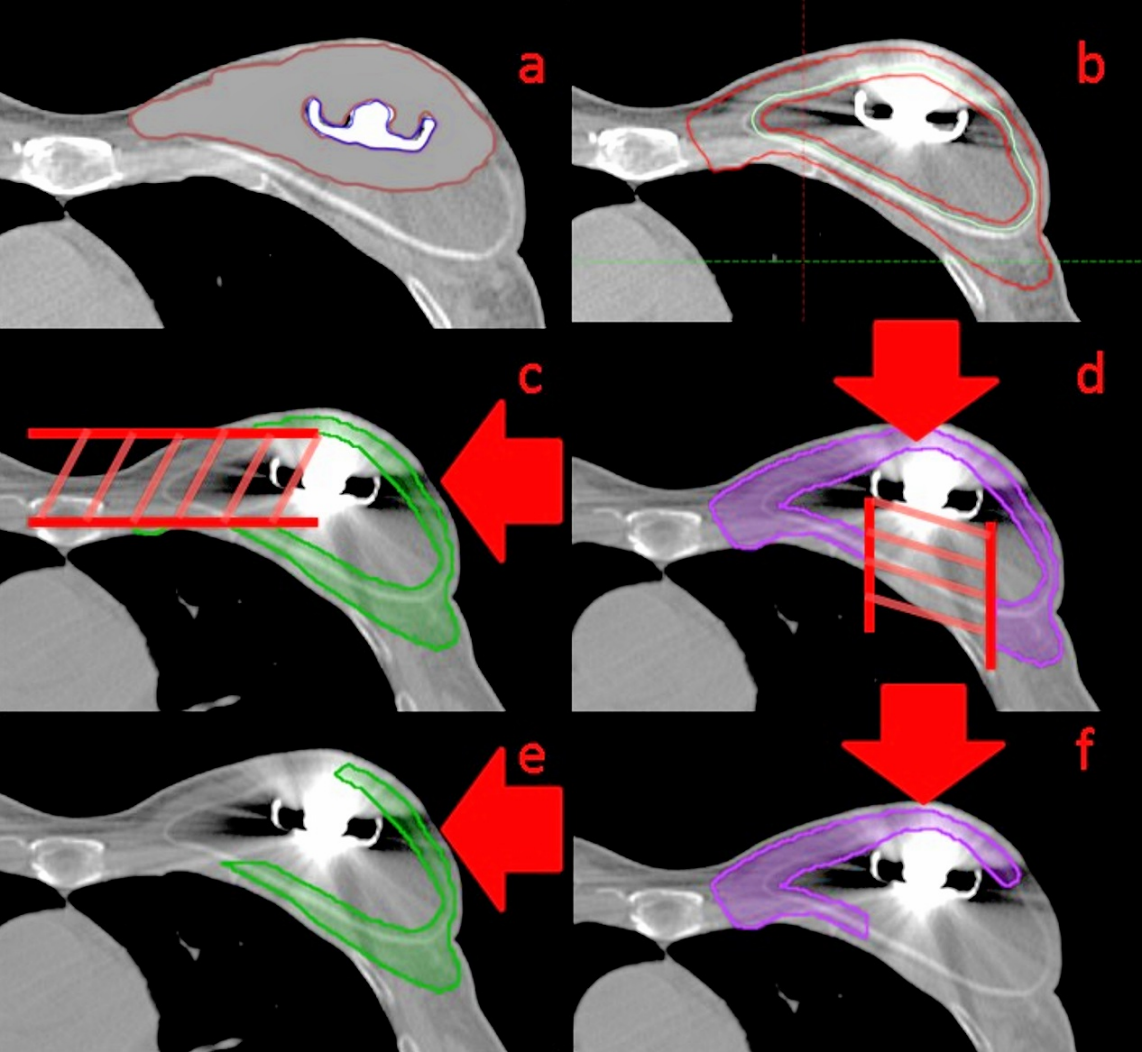
(a) The artifact override structure to account for CT artifact caused by the metal. (b) The contour of the implant (blue) and of the optimization target (red) which excludes all but a 0.3 cm rind of the implant. This is the target which is used in the optimization to apply prescription coverage. The field-specific targets for the lateral (c) and anterior (d) beams after excluding the section of the optimization target that is distal to the metal port with respect to the beam direction (indicated with red arrow). After defining a 3 cm overlap region through the center of the target where both beams will contribute dose, the final field-specific targets for planning for the lateral (e) and the anterior (f) beams are generated by excluding the section of the target beyond the overlap region with respect to the beam angle (field direction indicated with red arrow).

The image artifact produced by the metal port within the chestwall tissue and implant was contoured, and set the relative proton stopping power to 1.0 (water) (Figure 2a). The port itself (containing a neodymium magnet) was generously contoured using an auto-contouring, threshold relative proton stopping power of 1.4 for avoidance in planning. A 5-mm expansion of the port was created to aid in development of the field-specific targets.

Implant as non-target structure

A plan optimization target (CTV-opt) was created from the combined chest wall and nodal CTVs by first contouring the entire implant as a separate structure, then shrinking the implant structure by 3 mm from the tissue-implant interface, and subtracting the structure from the combined CTV (Figure 2b). The purpose of this subtraction was to allow the optimizer to remove the target dose requirement within the implant since there was no disease or normal tissue within the implant itself. This allows for flexibility in the optimization. The 3 mm border of the implant included in the optimization allows for setup shifts or distortion of the balloon.

Beam angle selection

Two beams are used to cover the target. One beam is from anterior direction, which has a more direct access to the axillary, internal mammary, and supraclavicular nodes, along with the medial and anterior chestwall tissues. A second left-lateral beam was selected, which allows direct access to the lateral portion of the target volume. The selection of complementary beam angles is critical to cover the entire target volume while avoiding the metal port. Field-specific targets were created based on the beam angles to limit the spots from each angle from traveling through to distal side of the metal. The necessary separation between the beam angles will depend on the distance from the posterior side of the port to the tissue beyond the implant. In this case, the distance was relatively small (1 cm); therefore, an 90-degree beam separation was necessary to allow for some overlap of field-specific targets, and to uniformly cover the targeted area posterior to the port. For larger implants, the lateral beam could be angled more toward en face as an anterior oblique beam.

Field-specific target

The field-specific targets are a copy of the CTV-opt structure that is subsequently cropped from the area distal to the 5 mm expansion of the port in the beam direction (Figure 2c, 2d). The 5 mm gap is used to minimize the impact of spot tails partially passing through the metal. The spot size in this plan is on the order of 8 mm to 9 mm for one sigma. The axillary and supraclavicular nodes are excluded from the lateral beam target to avoid having the beam pass through the shoulder area, thus the axillary and supraclavicular nodes are treated with the medial beam alone. A 3 cm overlap region is then defined through the middle of the target where both beams can send spots, allowing a smeared gradient match between the two fields. The portion of the field-specific target on the opposite side of the overlap region with respect to the beam angle is cropped out to create the final field-specific target (Figure 2e, 2f). The purpose of removing the contralateral portion of the target is to treat only the more en face portion of the target from each beam, and avoid sending spots to the portion of the target that is tangential to the beam, which would result in spots streaking into normal tissue (such as the contralateral chest wall).

Multi-field optimization

Eclipse™ treatment planning system (Varian Medical Systems, Palo Alto, California, USA) was used for planning. A multi-field optimization was used, which uses both beams simultaneously to optimize the full dose to the true target (CTV minus the inside of an implant). Although each beam only allows proton beam spots to be allocated to its own target volume (field-specific target), the optimization is done to the combined target. The dose in target volume that is not covered by the other beam will be solely responsible by one treatment field. The dose in the overlapped target region will be contributed by both treatment fields. Robust objectives were used for target coverage, considering 0.3 cm isocenter shifts combined with 3.5% shifts in relative proton stopping power. Field-independent isocenter shifts in the lateral direction were also entered into the robust optimization to allow for inverse optimization of the smeared gradient match between the two fields in the overlap region. Because breast target is shallow, the use of the range shifter is necessary for proton beams when treating superficial target. Range shifters degrade the proton beam and generate a relatively large pencil beam, which works well in the overlapping region because it creates a smooth dose gradient in the junction region that transits from one field to the other.

Robustness evaluation

To analyze the plan robustness with respect to setup error and systematic range uncertainty, the nominal plan is re-calculated with ±0.3 cm isocenter shifts applied in the x-, y-, and z-directions, combined with ±3.5% shift in relative proton stopping power. In order to evaluate the plan sensitivity to the construction of the metal port, the override of the contoured port structure was varied using proton relative stopping powers of 1, 2 and 3.38 (the largest value in our calibration curve). The plan was considered robust if the D95% was within 5% of the planned target coverage.

To analyze the plan robustness of the gradient in the field overlap region with respect to an overlap or gap between the fields, the individual fields of the nominal plan were independently shifted ±0.3 cm in the x-direction. The D98% for coverage and D1cc were compared with the nominal plan.

Photon plan comparison

An independent photon plan was generated from the referring photon therapy center in order to compare dose-volume indicators achievable between the planning techniques. The photon plan used three fields, with the internal mammary nodes treated in the chestwall tangent fields.

Planning results

**Figure 3:**
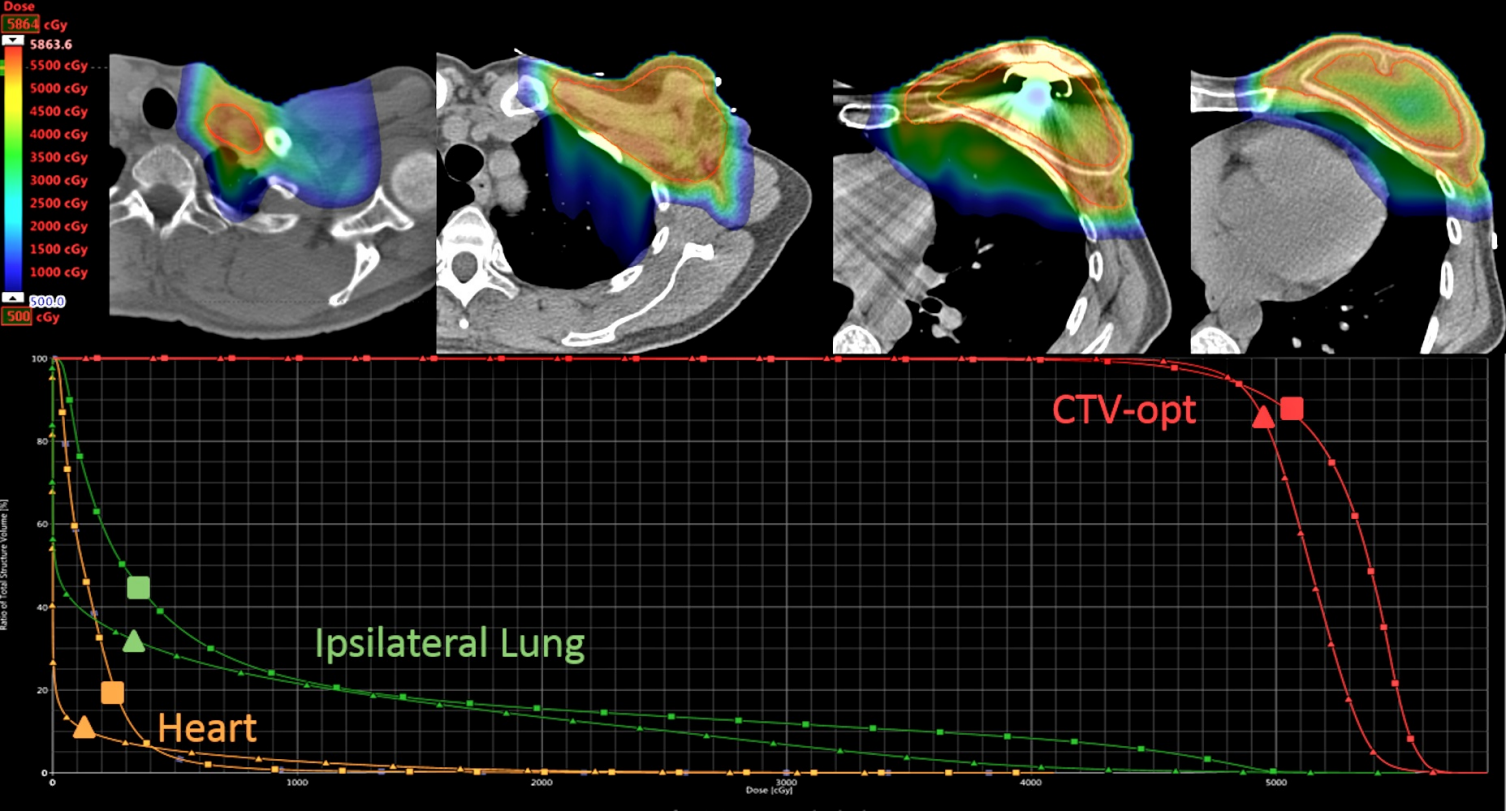
Dose distribution for the proton plan (above) with a dose-volume histogram comparison of the optimization target, ipsilateral lung and heart doses (below) comparing the proton (triangle) and photon (square) plans.

A comparative proton plan was generated using the field-specific multi-field optimization technique. Dose color wash, target coverage and OAR dose-volume histogram results are shown in Figure 3 with dose constraint results summarized in Table 1. The independent photon plan dose-volume histograms are overlaid for comparison. The only constraint the plan was unable to meet was the V105% and D1cc constraints for CTV-opt; however, the D1cc was within 1% of the constraint. The resulting nominal D95% target coverage for CTV-opt was 95.3%.

**Table 1:** Dose constraints and plan results

Structure	Dose Constraint	Proton Plan Result	Photon Plan Result
CTV-opt	D95% > 95%	D95% = 95.3%	D95% = 95.0%
CTV-opt	V105% < 10%	V105% = 18.5%	V105% = 66.9%
CTV-opt	D1cc < 110%	D1cc = 111.0%	D1cc = 112.5%
Ipsilateral Lung	V20Gy < 50%	V20Gy = 13.3%	V20Gy = 15.4%
Ipsilateral Lung	V5Gy < 65%	V5Gy = 28.2%	V5Gy = 35.6%
Contralateral Lung	V5Gy < 10%	V5Gy = 0.5%	V5Gy = 0.0%
Heart	V20Gy < 5%	V20Gy = 0.5%	V20Gy = 0.1%
Heart	Dmean < 4Gy	Dmean = 0.8Gy	Dmean = 1.7Gy
Contralateral Chestwall	V2Gy < 5%	V2Gy = 1.3%	V2Gy = 2.3%

Robustness

The worst-case target coverage considering 0.3 cm isocenter shifts combined with 3.5% shifts of relative proton stopping power was 90.1% which was within 5% of the planned target coverage. With the port contour set to a relative stopping power of 1, 2 and 3.38, the resulting target coverage was 95.5%, 95.4% and 95.0% respectively, all within 1% of planned target coverage.

**Figure 4:**
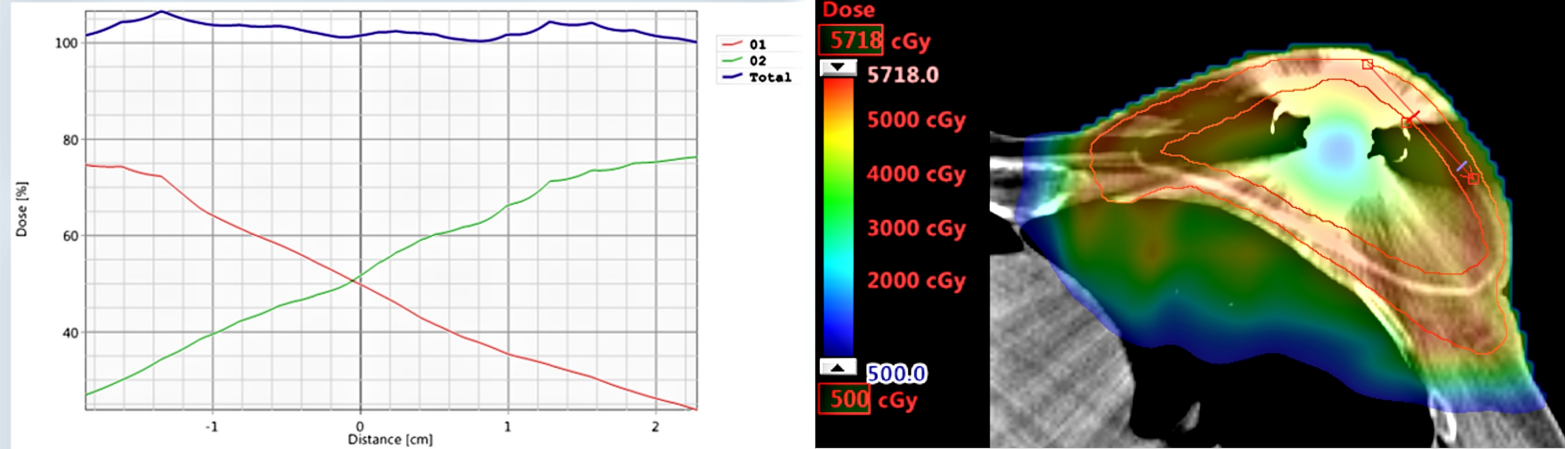
A dose profile through the overlap region between the two field-specific targets showing a smooth junction dose gradient between the two fields.

A smooth dose gradient was obtained in the overlap region between the fields using robust optimization objectives for individual field shifts. An example dose profile between the fields is shown in Figure 4. Independently shifting the field isocenters by ±0.3 cm in the x-direction only resulted in a maximum change of 0.3% for D98% and less than 0.1% in the D1cc for CTV-opt.

Photon plan comparison results

**Figure 5:**
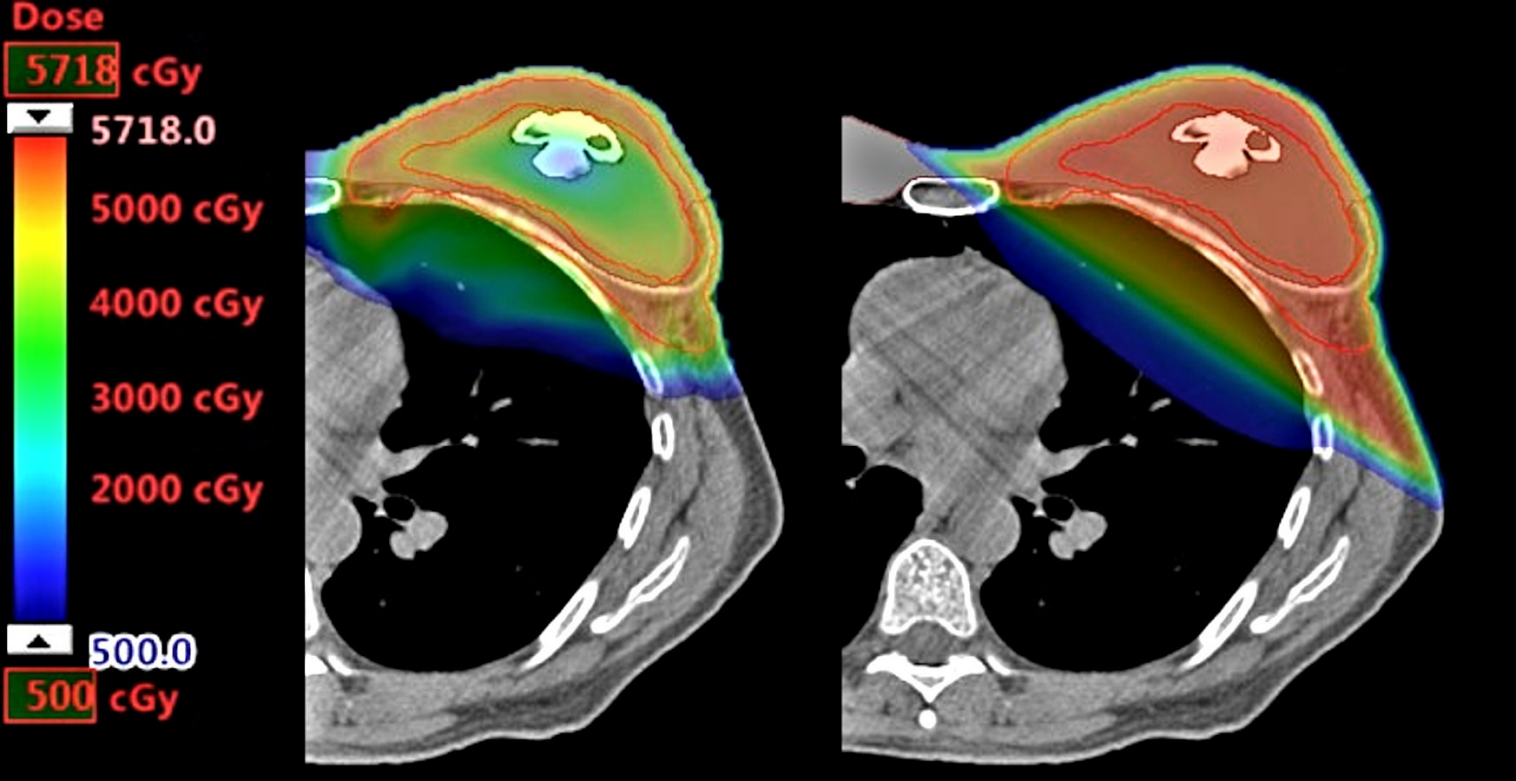
A dose color wash comparison between the proton plan (left) and photon plan (right).

The photon plan results are listed for comparison with the proton results in Table 1. The photon plan was also unable to meet V105% and D1cc constraints for the CTV-opt target if required to meet coverage of D95% by 95% of prescription, but otherwise met the OAR constraints. The V105% for the photon plan was 48.4% higher than the proton plan. Ipsilateral lung V5Gy and V20Gy, contralateral chestwall V2Gy, and the mean dose to the heart were lower in the proton plan. The V20Gy for the heart, and V5Gy for the contralateral lung were very low for both plans. A side-by-side comparison of the dose color wash for the two plans is shown in Figure 5.

## Discussion

This work presents a novel method of treatment planning using multi-field optimization to achieve a robust pencil beam scanning proton plan for breast cancer patients with implants containing a metal port. Through the use of field-specific targets that prevent proton beams from passing through the metal, this method allows treatment of a patient with a metal port without needing detailed information regarding the construction of the port or accurate, detailed contouring of the port components. This may be especially useful and practical when patients from outside facilities are referred for proton therapy, as obtaining information about their implant may be challenging. Our approach indicates that we can achieve target dose coverage goal while avoiding the metal port. It also retains the necessary robustness in the presence of setup errors or proton range uncertainties.

Until recently, the presence of a metal port in a breast cancer patient was considered to be a contraindication for the use of proton therapy. Mutter, et al. had proposed the first technique for treating these patients with intensity modulated proton therapy, allowing the proton beam to pass through the metal port while accounting for its measured stopping power in the plan calculation. They verified the accuracy of the plan calculation using Monte Carlo simulation. Their initial clinical results are promising and show acceptable skin toxicities [9]. Extending this technique to all patients would require measurement and verification for each type of port.

The example patient plan shown here represents a challenging case for proton therapy due to the thin target and the small distance between the posterior side of the metal port and the target. However, the planning technique was able to maintain clinically acceptable target coverage. With a thicker target, dose homogeneity is easier to achieve. Additionally, with a thicker implant, the angle of separation between the beams can be reduced so that both beams are more en face to the breast. At the same time, this patient data represents a best-case scenario for photon therapy, due to plan calculation on a deep inspiration breath hold scan, which has been shown to reduce lung and heart dose when treating with photons [10]. Deep inspiration breath hold does not similarly reduce heart and lung dose for proton plans due to the en face direction of the proton beams. This may be one of the reasons that the difference in ipsilateral lung and heart doses was not more drastic. Breath hold techniques may reduce the effect of breathing motion on the plan, but respiratory motion has been shown to have minimal impact on en face-type proton plans [11]. The selection of this patient with deep inspiration represents the best-case anatomical scenario for photon therapy and perhaps the worst-case scenario for proton therapy (due to the thin target). In our experience of over 15 patients planned with this approach, the dosimetric benefits for proton therapy are usually greater than the results reported here. Further analysis of individual heart vessel doses for a larger group of patients is ongoing.

## Conclusions

We demonstrated that a field-specific IMPT optimization technique can be used for planning breast cancer patients with tissue expanders. The technique is based on the principle of avoiding sending proton beams through the metal port of the tissue expander, which minimizes the risk of uncertainties in estimating proton stopping power for unknown metals. We also demonstrated that the planning technique is robust to setup uncertainties and proton range uncertainties, despite the use of multi-field IMPT optimization approach.
